# Progress Toward Poliomyelitis Eradication — Worldwide, January 2022–December 2023

**DOI:** 10.15585/mmwr.mm7319a4

**Published:** 2024-05-16

**Authors:** Keri Geiger, Tasha Stehling-Ariza, John Paul Bigouette, Sarah D. Bennett, Cara C. Burns, Arshad Quddus, Steven G.F. Wassilak, Omotayo Bolu

**Affiliations:** ^1^Epidemic Intelligence Service, CDC; ^2^Global Immunization Division, Global Health Center, CDC; ^3^Polio Eradication Department, World Health Organization, Geneva, Switzerland; ^4^Division of Viral Diseases, National Center for Immunization and Respiratory Diseases, CDC.

**Summary**

**What is already known about this topic?**

Afghanistan and Pakistan are the remaining countries with endemic transmission of wild poliovirus (WPV); however, multiple countries and regions are experiencing circulating vaccine-derived poliovirus (cVDPV) outbreaks.

**What is added by this report?**

Although the number of WPV cases in Afghanistan and Pakistan decreased during 2023, environmental surveillance detected WPV transmission outside known reservoir areas. Eight new countries reported cVDPV outbreaks, indicating a wider geographic spread of cVDPVs in 2023 compared with 2022.

**What are the implications for public health practice?**

To interrupt poliovirus transmission, a renewed focus on increasing routine immunization coverage in endemic areas and implementing higher quality supplementary immunization activities is necessary.

## Abstract

In 1988, poliomyelitis (polio) was targeted for eradication. Global efforts have led to the eradication of two of the three wild poliovirus (WPV) serotypes (types 2 and 3), with only WPV type 1 (WPV1) remaining endemic, and only in Afghanistan and Pakistan. This report describes global polio immunization, surveillance activities, and poliovirus epidemiology during January 2022–December 2023, using data current as of April 10, 2024. In 2023, Afghanistan and Pakistan identified 12 total WPV1 polio cases, compared with 22 in 2022. WPV1 transmission was detected through systematic testing for poliovirus in sewage samples (environmental surveillance) in 13 provinces in Afghanistan and Pakistan, compared with seven provinces in 2022. The number of polio cases caused by circulating vaccine-derived polioviruses (cVDPVs; circulating vaccine virus strains that have reverted to neurovirulence) decreased from 881 in 2022 to 524 in 2023; cVDPV outbreaks (defined as either a cVDPV case with evidence of circulation or at least two positive environmental surveillance isolates) occurred in 32 countries in 2023, including eight that did not experience a cVDPV outbreak in 2022. Despite reductions in paralytic polio cases from 2022, cVDPV cases and WPV1 cases (in countries with endemic transmission) were more geographically widespread in 2023. Renewed efforts to vaccinate persistently missed children in countries and territories where WPV1 transmission is endemic, strengthen routine immunization programs in countries at high risk for poliovirus transmission, and provide more effective cVDPV outbreak responses are necessary to further progress toward global polio eradication.

## Introduction

Since the establishment of the Global Polio Eradication Initiative (GPEI) in 1988, wild poliovirus (WPV) types 2 and 3 have been eradicated, and WPV type 1 (WPV1) remains endemic only in Afghanistan and Pakistan. In November 2021, a WPV1 outbreak genetically linked to Pakistan was detected in the southeastern African country of Malawi and spread to neighboring Mozambique in 2022 (*1*). Circulating vaccine-derived polioviruses (cVDPVs) can emerge and cause outbreaks in underimmunized populations when attenuated oral poliovirus vaccine (OPV) strains undergo prolonged person-to-person transmission that allows genetic mutation and recombination to occur, resulting in a reverted poliovirus with the ability to cause paralysis (*2*).

Polio eradication relies on achieving high population immunity to poliovirus through childhood routine immunization (RI) programs and supplementary immunization activities (SIAs) (vaccination campaigns). Since May 2016, bivalent OPV (bOPV), containing Sabin strains type 1 and 3 (instead of the trivalent OPV [tOPV] containing types 1, 2, and 3), and ≥1 dose of injectable, inactivated poliovirus vaccine (IPV, containing types 1, 2, and 3) have been used for RI programs in all OPV-using countries. RI and SIAs using bOPV raise immunity against poliovirus types 1 and 3, protecting against WPV1 spread in countries with endemic transmission and against emergence of cVDPV type 1 or type 3 outbreaks. Monovalent OPV Sabin-strain type 2 (mOPV2) and novel OPV2 (nOPV2) vaccine, a more genetically stable OPV2 vaccine, are reserved for cVDPV2 outbreak response (*3*,*4*). Used in SIAs since March 2021 under emergency use listing (EUL), nOPV2 received World Health Organization (WHO) prequalification in December 2023. Since the EUL, nOPV2 vaccine has been primarily used in response to cVDPV2 outbreaks; mOPV2 and tOPV were last used during SIAs in March 2023.

The GPEI goal to interrupt all types of poliovirus transmission was not achieved in December 2023 (*5*). This report summarizes the status of polio eradication during January 1, 2022–December 31, 2023, and updates previous reports (*1*,*6*).

## Methods

Surveillance and virologic data were gathered from WHO’s global Polio Information System (POLIS).* Immunization coverage data were retrieved from WHO’s Immunization Dashboard and represent estimates made by WHO and UNICEF based on administrative reporting (i.e., vaccine doses administered divided by the estimated target population) and survey data (*7*). A cVDPV outbreak was defined as a case of acute flaccid paralysis (AFP) with laboratory-confirmed cVDPV and evidence of community transmission or at least two positive cVDPV isolations from environmental sampling taken from two different sites without overlapping catchment areas, or from the same site, with ≥1 month between isolations.^†^ Descriptive analyses were conducted using R software (version 4.3.1; R Foundation). This activity was reviewed by CDC, deemed not research, and was conducted consistent with applicable federal law and CDC policy.^§^

## Results

### Poliovirus Vaccination

In 2022, WHO recommended that all children worldwide be vaccinated against all polio types with ≥3 IPV doses (for countries using an IPV-only schedule) or ≥3 bOPV doses plus 2 IPV doses, in countries using a combined OPV-IPV schedule.^¶^ The estimated global RI coverage with ≥3 doses of IPV or OPV (Pol3) by age 12 months was 84% in 2022, compared with 81% in 2021 and 83% in 2020 (*7*). Coverage during 2020–2022 remained below the 85%–87% range reported annually during 2014–2019 (*7*), before COVID-19 pandemic–associated disruptions in RI programs. In 2022, estimated coverage with 1 full dose or 2 fractional doses of IPV (IPV1; one fifth of a full IPV dose administered intradermally) was 84%, an increase from 80% in 2020 and 2021 and above the pre–COVID-19 pandemic high of 83% in 2019 (*7*). In 2022, estimated national coverage with Pol3 and IPV1 was 76% and 71%, respectively, in Afghanistan and 85% and 90%, respectively, in Pakistan (*7*). Subnational administrative data indicate much lower RI polio vaccination coverage in WPV1 reservoir areas.

In 2023, a total of 119 SIAs were conducted in 30 countries worldwide. Approximately 675 million doses of bOPV were administered in 10 countries (including 114 million doses in Afghanistan and 264 million in Pakistan), 10 million doses of mOPV2 were administered in Sudan, 524 million doses of nOPV were administered in 24 countries, and 8 million doses of tOPV were administered in two countries (Somalia and Yemen). Although nOPV2 is the preferred vaccine for cVDPV2 outbreak response because it is more genetically stable than the Sabin strain in tOPV or mOPV2 (which is more likely than the nOPV2 strain to revert to neurovirulence), production by a single manufacturer has resulted in vaccine supply shortages.

### Poliovirus Surveillance

Case-based surveillance for AFP in persons aged <15 years is the primary means for detecting WPV and cVDPV transmission. Environmental surveillance (ES), the testing of sewage samples for poliovirus, supplements AFP surveillance and can detect poliovirus circulation in the absence of AFP cases. Reported AFP cases are confirmed if poliovirus is isolated in a stool specimen at one of the 144 WHO-accredited laboratories in the Global Polio Laboratory Network (*8*). The two indicators used to monitor polio surveillance performance are the nonpolio AFP rate (a rate of two or more cases per 100,000 persons aged <15 years indicates sufficiently sensitive AFP surveillance) and stool adequacy (collection of two stool specimens of sufficient quality ≥24 hours apart and within 14 days of paralysis onset and received in good condition at a WHO-accredited laboratory via reverse cold chain for >80% of AFP cases). In 2023, eight** of 28 (29%) countries at high risk for poliovirus spread^††^ failed to meet global AFP surveillance indicator targets at the national level. In 2023, a total of 15,886 ES samples from 1,462 sites in 68 countries were tested, representing an increase from 14,498 samples (10% increase) from 1,117 sites (31% increase) in 69 countries in 2022.

### Reported Poliovirus Cases and Isolations

**Countries reporting WPV cases and isolations.** In 2023, Afghanistan and Pakistan reported six WPV1 cases each, compared with two in Afghanistan and 20 in Pakistan in 2022 (Table 1) (Figure) (*9*,*10*). In Afghanistan, all six reported cases in 2023 were from Nangarhar province, and the two cases reported in 2022 were from Paktika and Kunar provinces. These three provinces are located along the country’s eastern border with Pakistan and have security challenges (*9*).

**TABLE 1:** Number of poliovirus cases and isolations detected through environmental surveillance of wild poliovirus type 1 and circulating vaccine-derived polioviruses — worldwide, January 1, 2022–December 31, 2023*

Country or territory (poliovirus type)	No. of poliovirus cases				No. of isolations of poliovirus through ES			
	2022		2023		2022		2023	
	WPV1	cVDPV	WPV1	cVDPV	No. of samples	No. (%) positive	No. of samples	No. (%) positive
**Reporting WPV1** ^†^								
Afghanistan	2	0	6	0	698	22 (3)	521	62 (12)
Pakistan	20	0	6	0	1,199	36 (3)	2,202	124 (6)
Mozambique (1,2)	8	26	0	5	133	0 (—)	211	0 (—)
**Reporting cVDPV** ^†^								
Algeria (2)	0	3	0	0	76	18 (24)	142	27 (19)
Angola (2)	0	0	0	0	128	0 (—)	122	1 (1)
Benin (2)	0	13	0	3	109	9 (8)	116	4 (3)
Botswana (2)	0	0	0	0	22	5 (23)	135	5 (4)
Burkina Faso (2)	0	0	0	3	151	0 (—)	154	0 (—)
Burundi (2)	0	1	0	1	34	7 (21)	50	13 (26)
Cameroon (2)	0	3	0	0	410	0 (—)	597	13 (2)
Canada (2)	0	0	0	0	2	2 (100)	0	0 (—)
Central African Republic (2)	0	6	0	14	212	9 (4)	222	1 (0.5)
Chad (2)	0	44	0	55	86	7 (8)	92	3 (3)
Congo (1)	0	1	0	0	238	0 (—)	246	2 (1)
Côte d’Ivoire (2)	0	0	0	6	157	3 (2)	262	43 (16)
Democratic Republic of the Congo (1,2)	0	522	0	223	327	13 (4)	441	37 (8)
Djibouti (2)	0	0	0	0	11	11 (100)	43	0 (—)
Egypt (2)	0	0	0	0	641	6 (1)	696	11 (2)
Eritrea (2)	0	1	0	0	0	0 (—)	0	0 (—)
Ethiopia (2)	0	1	0	0	31	0 (—)	29	0 (—)
Ghana (2)	0	3	0	0	197	20 (10)	167	0 (—)
Guinea (2)	0	0	0	47	123	0 (—)	146	19 (13)
Indonesia (2)	0	1	0	6	143	0 (—)	107	1 (1)
Israel (2,3)	0	1	0	1	82	80 (98)	0	0 (—)
Kenya (2)	0	0	0	8	200	0 (—)	227	8 (4)
Madagascar (1)	0	16	0	24	668	156 (23)	551	99 (18)
Malawi (1,2)	0	4	0	0	353	0 (—)	276	1 (0.4)
Mali (2)	0	2	0	15	46	0 (—)	67	6 (9)
Mauritania (2)	0	0	0	1	82	0 (—)	62	3 (5)
Niger (2)	0	16	0	2	301	14 (5)	501	4 (1)
Nigeria (2)	0	48	0	87	2,242	82 (4)	1,598	84 (5)
Occupied Palestinian Territory (3)	0	0	0	0	9	9 (100)	0	0 (—)
Senegal (2)	0	0	0	0	286	0 (—)	321	1 (0.3)
Somalia (2)	0	5	0	8	11	4 (36)	230	9 (4)
South Sudan (2)	0	0	0	3	49	0 (—)	230	0 (—)
Sudan (2)	0	1	0	0	154	1 (1)	89	5 (6)
Tanzania (2)	0	0	0	2	151	0 (—)	210	6 (3)
Togo (2)	0	2	0	0	87	0 (—)	96	0 (—)
United Kingdom (2)	0	0	0	0	26	6 (23)	1	0 (—)
United States (2)	0	1	0	0	31	0 (—)	0 (—)	0 (—)
Yemen (2)	0	160	0	8	34	26 (76)	33	10 (30)
Zambia (2)	0	0	0	1	117	3 (3)	254	2 (12)
Zimbabwe (2)	0	0	0	1	0	0 (—)	55	17 (31)
**Total**	**30**	**881**	**12**	**524**	**9,606**	**549 (6)**	**10,782**	**621 (5)**

**Abbreviations:** cVDPV = circulating vaccine-derived poliovirus; ES = environmental surveillance; WPV1 = wild poliovirus type 1.

* Data current as of April 10, 2024.

^†^ Countries listed are only those with reported poliovirus cases or isolations.

**FIGURE:**
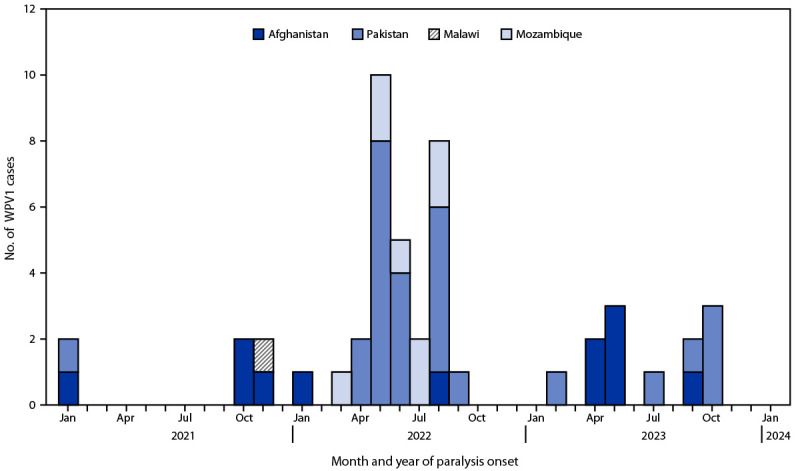
Wild poliovirus type 1 cases — worldwide, January 2021–December 2023 **Abbreviation: **WPV1 = wild poliovirus type 1.

Four of the six 2023 WPV1 cases in Pakistan were from Khyber Pakhtunkhwa province, a northern province bordering Afghanistan, and two cases were from the southern Sindh province, specifically Karachi city. In 2022, WPV1 cases were confined to Khyber Pakhtunkhwa province, which is an area known for security and health access challenges (*10*).

In 2023, among 521 environmental samples tested for poliovirus from Afghanistan, 62 (12%) were positive for WPV1, compared with 22 of 698 (3%) samples tested in 2022 (Table 1). Whereas this finding represents a 25% decrease in the total number of samples tested from 2022 to 2023, the percentage that tested positive quadrupled. ES isolates were identified in eight provinces, including seven that did not report any WPV1 cases identified by AFP surveillance. Seven of the eight provinces with positive ES isolates were near the border with Pakistan.

In Pakistan, 124 of 2,202 (6%) environmental samples tested for poliovirus in 2023 were positive for WPV1, representing an 84% increase compared with those tested in 2022 (1,199), and a doubling of the 3% positivity rate (36 of 1,199) in 2022. These ES isolates came from five provinces covering most of the country, including three that did not report any WPV1 cases identified by AFP surveillance. Within Afghanistan and Pakistan, the number of provinces with WPV1 environmental isolates nearly doubled from seven in 2022 to 13 in 2023 (Table 2).

**TABLE 2:** Number of administrative areas with poliovirus detections through acute flaccid paralysis surveillance and environmental surveillance, by poliovirus type and administrative area — worldwide, January 1, 2017–December 31, 2021*

Poliovirus type/ Administrative area	No. of areas						
	2017	2018	2019	2020	2021	2022	2023
**WPV1**							
Countries	2	2	3	2	3	3	2
Provinces	15	14	18	25	9	8	13
Districts	37	52	94	116	28	26	48
**cVDPV (any type)**							
Countries	4	9	22	33	32	32	32
Provinces	8	36	106	205	145	137	148
Districts	16	75	230	598	447	386	328
**cVDPV1**							
Countries	0	2	5	4	2	5	3
Provinces	0	10	7	6	9	17	19
Districts	0	16	13	18	19	45	45
**cVDPV2**							
Countries	4	7	19	30	31	29	31
Provinces	8	26	103	200	137	123	133
Districts	16	59	223	581	429	357	294

**Abbreviations:** cVDPV = circulating vaccine-derived poliovirus; cVDPV1 = CVDPV type 1; cVDPV2 = cVDPV type 2; WPV1 = wild poliovirus type 1.

* Data are current as of April 10, 2024.

The 2021–2022 WPV1 outbreak in Africa included one WPV1 case in Malawi (November 2021) and eight cases in one province in Mozambique in 2022 (*1*). All Mozambique cases were genetically linked to the Malawi case and the Malawi case was linked to Pakistan. No WPV1 cases have been reported outside of Afghanistan and Pakistan since August 10, 2022.

**Countries reporting cVDPV cases and isolations.** The total number of cVDPV cases worldwide decreased 41% from 2022 (881 cases: 193 cVDPV1, 687 cVDPV2, and one cVDPV3 case) to 2023 (524 cases: 133 cVDPV1 and 391 cVDPV2) (Table 1), representing a 31% decrease in cVDPV1 cases and a 43% decrease in cVDPV2 cases. The Democratic Republic of the Congo and Mozambique each reported both cVDPV1 and cVDPV2 cases in 2022 and 2023. No country reported cVDPV3 in 2023, although one case was reported in Israel in 2022.

Although only 23 countries reported cVDPV polio cases in 2022 and 2023, active cVDPV outbreaks confirmed only through ES isolation were reported in an additional nine countries each year (Table 1). Among these 32 countries, eight^§§^ that reported cVDPVs in 2022 stopped their outbreaks and did not report cVDPVs in 2023. Among the 32 countries reporting cVDPVs in 2023,^¶¶^ eight countries*** that had not reported any cVDPV cases or isolates in 2022 had new outbreaks (Table 1) (Table 2). Despite completing planned outbreak response SIAs, 24 countries^†††^ with cVDPV detections in 2022 failed to stop their outbreaks, indicating that enough children are persistently missed during the SIAs to sustain cVDPV transmission.

## Discussion

Global WPV1 eradication efforts remain hindered by continuing transmission in areas of Afghanistan and Pakistan with ongoing security issues that limit health service access. In Afghanistan and Pakistan, WPV cases were detected in only a total of three provinces in 2023. However, in both countries, ES detected WPV1 across multiple provinces remote from the location of WPV1 cases, indicating widespread WPV1 transmission, with ongoing local transmission in some areas. Efforts to increase SIA quality and access more children, synchronize campaigns at the Pakistan-Afghanistan border, and strengthen active AFP surveillance aimed at disrupting WPV1 transmission in both countries are continuing.

Ongoing cVDPV outbreaks pose additional challenges to global polio eradication efforts. In 2023, eight countries reported new cVDPV outbreaks and cVDPV transmission continued in 24 countries with outbreaks ongoing since 2022, indicating that children continue to be missed during SIA vaccination rounds. Substantial improvement in response efforts is needed. However, with a single nOPV2 manufacturer, new cVDPV2 outbreaks, and uninterrupted transmission, nOPV2 vaccine supply has been insufficient to meet outbreak response needs. Interruptions in nOPV2 vaccine availability during 2024 will delay SIAs for cVDPV2 outbreak responses, risking further spread. Continued focus on high-quality, appropriately scaled SIA responses is critical to interrupting cVDPV2 circulation. In countries with prolonged cVDPV outbreaks, serious access issues, and security concerns, alternative strategies are required to reach missed children.

### Limitations

The findings in this report are subject to at least two limitations. First, administrative vaccination coverage estimates are based on population estimates that might be inaccurate in areas lacking recent census data or those with substantial population migration. As a result, RI and SIA coverage might be over- or underestimated. Second, AFP surveillance results rely on accurately identifying AFP cases; the numbers presented in this report might include cases that do not meet the AFP case definition and exclude cases not reported to the program.

### Implications for Public Health Practice

Accelerating progress toward polio eradication will require reaching persistently missed children by implementing effective, innovative campaigns in areas with ongoing insecurity concerns, increasing community stakeholders’ engagement in vaccination efforts, and enhancing accountability at national and community levels, including frontline supervision. Ensuring sufficient vaccine supply for outbreak response practices and limiting the geographic spread of outbreaks into poliovirus-free areas are also needed. These improvements are critical to interrupting endemic and outbreak-associated poliovirus transmission and achieving the goal of global polio eradication.

## Footnotes

* POLIS is a centralized database that integrates polio case-based and environmental surveillance, with supplemental immunization activity data from all WHO regions. https://extranet.who.int/polis (Access is limited to members of GPEI partner organizations).

†
https://polioeradication.org/wp-content/uploads/2022/09/Standard-Operating-Procedures-For-Responding-to-a-Poliovirus-Event-Or-Outbreak-20220905-V4-EN.pdf

§ 45 C.F.R. part 46.102(l)(2), 21 C.F.R. part 56; 42 U.S.C. Sect. 241(d); 5 U.S.C. Sect. 552a; 44 U.S.C. Sect. 3501 et seq.

¶
https://www.who.int/publications/i/item/WHO-WER9725-277-300

** Angola, Botswana, Burundi, Indonesia, Niger, Papua New Guinea, Sudan, and Zambia.

†† Priority countries were selected for this report because of gaps in surveillance and vulnerability to poliovirus circulation, as determined in the WHO Global Polio Surveillance Action Plan, 2022–2024 (https://polioeradication.org/wp-content/uploads/2022/05/GPSAP-2022-2024-EN.pdf), updated June 2023. Countries include the following: *African Region* (21): Angola, Botswana, Burkina Faso, Burundi, Cameroon, Central African Republic, Chad, Democratic Republic of the Congo, Ethiopia, Guinea, Kenya, Madagascar, Malawi, Mali, Mozambique, Niger, Nigeria, South Sudan, Tanzania, Zambia, and Zimbabwe; *Eastern Mediterranean Region* (five): Afghanistan, Pakistan, Somalia, Sudan, and Yemen; *South-East Asia Region* (one): Indonesia; and *Western Pacific Region* (one): Papua New Guinea.

§§ Canada, Djibouti, Eritrea, Ethiopia, Ghana, Togo, United Kingdom, and United States.

¶¶ Algeria, Angola, Benin, Botswana, Burkina Faso, Burundi, Cameroon, Central African Republic, Chad, Congo, Côte d’Ivoire, Democratic Republic of the Congo, Egypt, Guinea, Indonesia, Israel, Kenya, Madagascar, Malawi, Mali, Mauritania, Mozambique, Niger, Nigeria, Senegal, Somalia, South Sudan, Sudan, Tanzania, Yemen, Zambia, and Zimbabwe.

*** Angola, Burkina Faso, Guinea, Kenya, Mauritania, South Sudan, Tanzania, and Zimbabwe.

††† Algeria, Benin, Botswana, Burundi, Cameroon, Central African Republic, Chad, Congo, Côte d’Ivoire, Democratic Republic of the Congo, Egypt, Indonesia, Israel, Madagascar, Malawi, Mali, Mozambique, Niger, Nigeria, Senegal, Somalia, Sudan, Yemen, and Zambia.
